# Waveform changes of laser speckle flowgraphy in the temporal optic nerve head and peripapillary atrophy after trabeculectomy in open-angle glaucoma

**DOI:** 10.1038/s41598-022-13989-2

**Published:** 2022-06-13

**Authors:** Makoto Sasaki, Tomomi Higashide, Satoshi Takeshima, Yuki Takamatsu, Yoshimi Manbo, Sachiko Udagawa, Kazuhisa Sugiyama

**Affiliations:** 1 Department of Ophthalmology, Kanazawa University Graduate School of Medical Sciences, Takara-mach13-1, Kanazawa, Ishikawa-ken 920-8641 Japan; 2 Department of Ophthalmology, Fukui-Ken Saiseikai Hospital, Fukui, Japan; 3 Department of Ophthalmology, Toyama Red Cross Hospital, Toyama, Japan; 4 Department of Ophthalmology, Toyama City Hospital, Toyama, Japan; 5 Department of Ophthalmology, Koseiren Takaoka Hospital, Takaoka, Japan

**Keywords:** Glaucoma, Eye abnormalities

## Abstract

A prospective study was conducted on 33 eyes of 33 patients with open-angle glaucoma who underwent trabeculectomy to investigate hemodynamic changes in the temporal optic nerve head (ONH) and peripapillary atrophy (PPA) after trabeculectomy. Laser speckle flowgraphy of ONH and PPA was performed at baseline and at 1, 3, and 6 months postoperatively. The waveforms of the mean blur rate in the tissue area (MT) in the temporal ONH, βPPA (with Bruch’s membrane), and γPPA (without Bruch’s membrane) were evaluated. Mean intra-ocular pressure (IOP) decreased from 19.1 ± 0.8 to 8.5–9.6 ± 0.7 mmHg at postoperative visits. The average MT in the βPPA region increased significantly at all postoperative time points, whereas those in the ONH and γPPA regions remained unchanged. The blowout score (BOS) increased significantly, and the resistivity index decreased significantly at all time points in all regions, which was associated with decreased IOP. The current study showed two novel findings: MT increased after trabeculectomy only in βPPA, where the choroid was present. IOP decrease-associated BOS increase occurred postoperatively in all regions, which indicates that IOP reduction may decrease vascular transmural pressure and contribute to stable blood flow uniformly, despite structural differences between the regions.

## Introduction

Among several factors involved in glaucomatous optic neuropathy (GON), peripapillary atrophy (PPA) is associated with the onset and progression of GON^1–5^. PPA is funduscopically classified into α- and β-zone; α-zone corresponds to the outer area with irregularity of pigmentation, and β-zone corresponds to the inner area with increased visibility of large choroidal vessels or sclera due to the atrophy of photoreceptors/retinal pigment epithelium (RPE) and closure of the choriocapillaris^2,3^. Notably, the β-zone PPA is relevant to GON^6,7^. Owing to the advances in optical coherence tomography (OCT) for detailed delineation of the deep structures including the choroid^8^, β-zone PPA is further classified based on the presence (βPPA) or absence (γPPA) of Bruch's membrane. Extension of βPPA has been linked to glaucoma progression, whereas γPPA, which is present inside the Bruch's membrane opening (BMO) and corresponds to the border tissue, is associated with myopic structural deformation in optic nerve head (ONH)^9,10^.

Several studies have reported changes in the blood flow in the fundus after trabeculectomy^11–22^. Takamatsu et al. showed that blood flow in the macula, measured using laser speckle flowgraphy (LSFG), significantly increased after trabeculectomy^22^. Tamaki et al. and Takeshima et al. reported that there was no change in tissue blood flow in ONH measured by LSFG after trabeculectomy^15,16^. Kim et al. reported an increase in the vessel density in the lamina cribrosa, as measured by OCT angiography after trabeculectomy^18^. They also showed that there was no change in the vessel density in the peripapillary area, whereas In et al. reported an increase in the vessel density in this region^18,20^. Postoperative blood flow changes may vary depending on the location and measurement device used. However, to the best of our knowledge, postoperative blood flow changes in PPA have not been addressed. Since PPA is closely associated with GON and its progression, it is worth investigating the changes in blood flow after trabeculectomy in the PPA region. In this study, we focused on the temporal quadrant of ONH, which has β and γPPA more frequently than the other quadrants, and disclosed the difference in the blood flow changes between ONH, βPPA, and γPPA after trabeculectomy in glaucomatous eyes.

## Results

The characteristics of the 33 eyes of 33 patients at baseline are shown in Table 1. The mean age was 69.3 ± 8.7 years, and 14 (42.4%) patients were male. Primary open-angle glaucoma was found in 24 eyes (72.7%), and the baseline mean deviation was − 20.1 ± 5.8 dB. Moreover, 15 subjects (45.5%) had systemic hypertension, and 11 (73.3%) were administered hypotensive agents. The mean preoperative Intraocular pressure (IOP) was 19.1 ± 6.0 mmHg, and the mean preoperative medication score was 4.0 ± 0.7. Additionally, six patients (18.2%) were administered systemic carbonic anhydrase inhibitors. Supplementary Table S1 shows the baseline values of parameters in the temporal ONH, βPPA, and γPPA.

**Table 1:** Patient demographics.

Age	69.3 ± 8.7 (54 to 85)
Sex, male/female	14/19
Diagnosis, POAG/XFG, eyes	24/9
**Systemic hypertension, cases (%)**	15 (45.5)
Oral antihypertensive agents, cases (%)	11 (33.3)
Oral calcium channel blockers, cases (%)	8 (24.2)
Oral angiotensin II receptor blockers, cases (%)	7 (21.2)
Oral β blockers, cases (%)	3 (9.1)
Diabetes mellitus, cases (%)	1 (3.0)
Axial length, mm	24.7 ± 1.1 (22.6 to 26.7)
Intraocular pressure, mm Hg	19.1 ± 6.0 (12 to 42)
Mean deviation, dB	− 20.1 ± 5.8 (− 8.4 to − 29.3)
Systolic blood pressure, mm Hg	127.4 ± 18.9 (90 to 166)
Diastolic blood pressure, mm Hg	69.2 ± 12.3 (49 to 95)
Mean arterial pressure, mm Hg	88.4 ± 14.5 (50.3 to 117.0)
Ocular perfusion pressure, mm Hg	39.9 ± 9.5 (17.6 to 57.0)
Pulse rate, beats/min	69.2 ± 10.5 (57.3 to 95.0)
Preoperative use of oral acetazolamide, eyes (%)	6 (18.2)
**No. of preoperative antiglaucoma medications, eyes**	4.0 ± 0.7
Prostaglandin analogues (%)	33 (100)
β antagonists (%)	29 (87.9)
Carbonic anhydrase inhibitors (%)	31 (93.9)
α-1 antagonist (%)	8 (24.2)
α-2agonist (%)	28 (84.9)
Rho kinase inhibitor (%)	2 (6.1)
**Previous intraocular surgery, eyes (%)**	
Cataract surgery	11 (33.3)
Trabeculotomy	5 (15.2)
Laser trabeculoplasty	1 (3.0)

*POAG* Primary open angle glaucoma, *XFG* exfoliation glaucoma.

The changes in IOP and ocular perfusion pressure (OPP) are shown in Supplementary Fig. S1. IOP was significantly decreased at all postoperative time points, from 19.1 ± 0.8 mmHg to 9.6 ± 0.7, 8.5 ± 0.7, and 9.5 ± 0.7 mmHg at 1, 3 and 6 months. OPP was significantly increased at all postoperative timepoints, from 39.9 ± 1.6 mmHg to 53.1 ± 1.7, 53.4 ± 1.7, and 53.6 ± 1.6 mmHg at 1, 3 and 6 months, respectively.

Supplementary Figure S2 shows the retinal thickness changes in the βPPA and γPPA regions, and choroidal thickness changes in the βPPA region in the temporal quadrant of ONH. In the βPPA region, the choroidal thickness was significantly increased at all time points after trabeculectomy, from 78.7 ± 6.8 μm to 90.1 ± 7.1, 90.8 ± 7.6 and 87.5 ± 8.4, while retinal thickness was significantly increased from 162.1 ± 5.7 to 168.8 ± 5.8 μm at 1 month. In the γPPA region, the retinal thickness significantly increased from 136.9 ± 8.3 to 147.5 ± 8.4 μm at 1 month.

**Figure 1:**
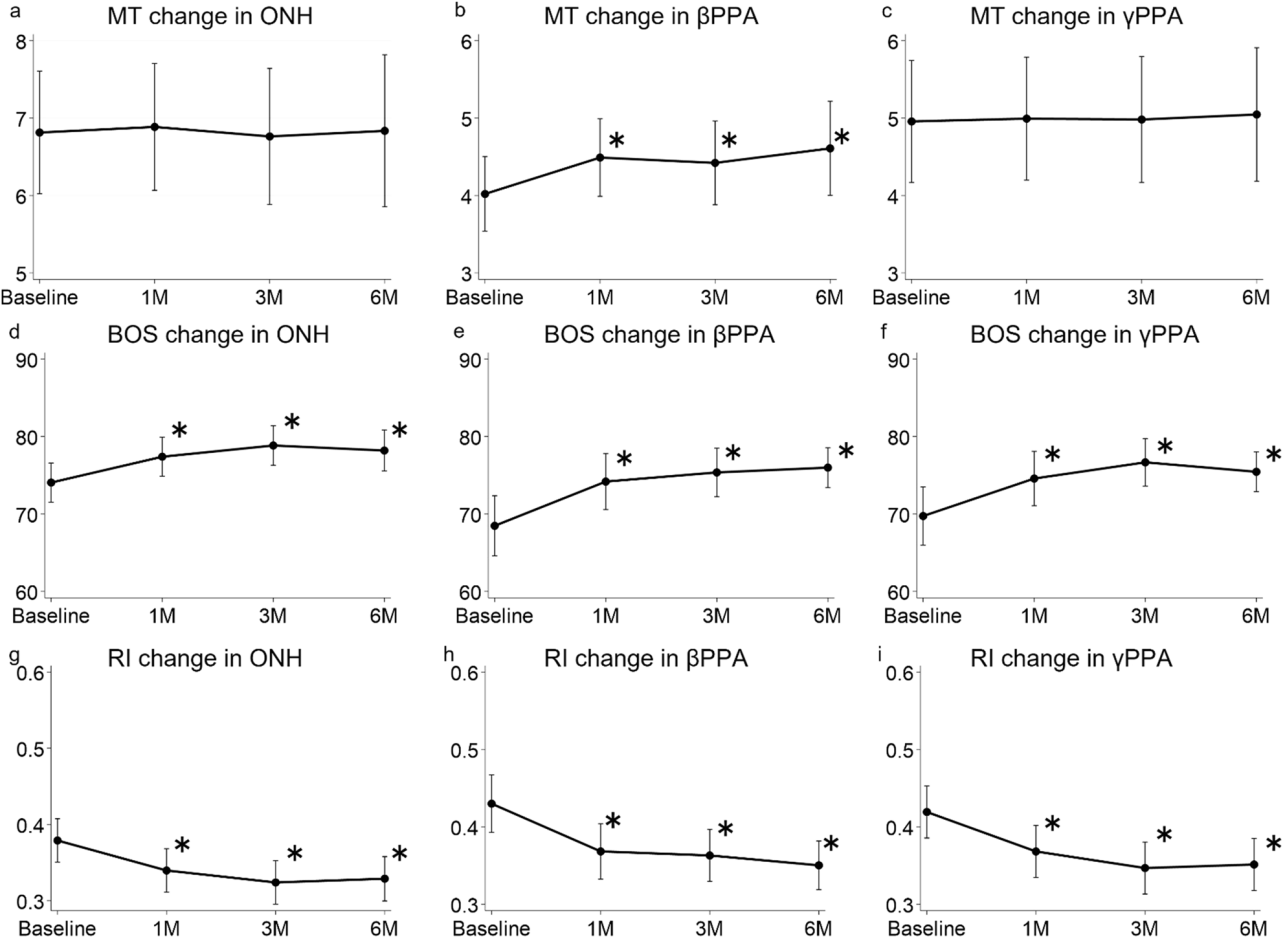
Changes in MBR waveform parameters in temporal ONH, βPPA, and γPPA after trabeculectomy. (**a–c**) MT in ONH, βPPA, and γPPA, respectively. (**d–f**) BOS in ONH, βPPA, and γPPA, respectively. (**g–i**) RI in ONH, βPPA, and γPPA, respectively. *P < 0.05. *MBR* mean blur rate, *MT* average MBR in the tissue area, *BOS* blowout score, *RI* resistivity index.

**Figure 2:**
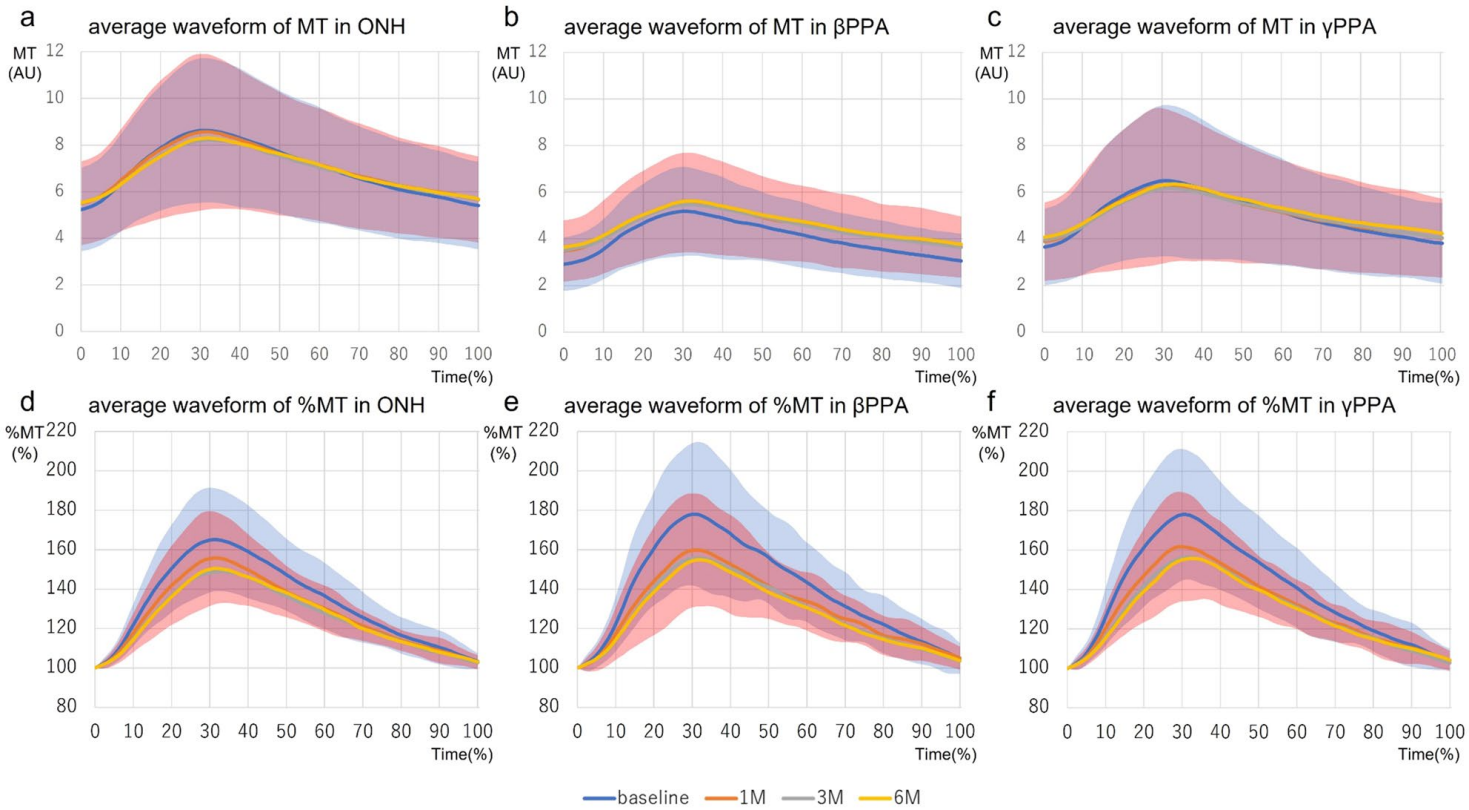
Changes in the average waveform of MT in temporal ONH, β PPA, and γPPA after trabeculectomy. The average waveform of MT in ONH (**a,d**), βPPA (**b,e**), and γPPA (**c,f**) at each time point was determined by normalizing the heartbeat duration of each patient. The horizontal axes represent the time normalized with one heartbeat duration of 100%. The vertical axes in figure (**a–c**) represent MT (AU). The vertical axes in figure (**d–f**) represent the relative MT (%) to the MBR at 0%. The light blue and light red shaded regions indicate the standard deviations of the average values at baseline and 1 month postoperatively, respectively.

Figure 1 shows the changes in the blood flow waveform parameters in the temporal ONH, βPPA, and γPPA regions. In the temporal βPPA region, the average MBR in tissue area (MT) significantly increased from 4.0 ± 0.2 to 4.5 ± 0.3, 4.4 ± 0.3, and 4.6 ± 0.3 at 1, 3 and 6 months postoperatively, respectively. However, no significant postoperative changes in MT were found in the temporal ONH and γPPA regions. Figure 2 shows the average waveform of MT in the ONH, β PPA, and γPPA regions. MT in βPPA increased throughout the duration of the heartbeat at all postoperative time points. The blood flow waveforms flattened after trabeculectomy in the ONH, βPPA, and γPPA regions. The blowout score (BOS) increased significantly, and the resistivity index (RI) decreased significantly at all postoperative time points in all measurement regions. The other parameters did not show significant postoperative changes in any region (Supplementary Fig. S3).

In the univariate analysis of MT change in the temporal βPPA region, worse baseline mean deviation and an increase in mean arterial pressure (MAP) and OPP were significantly associated with an increase in postoperative MT (Table 2). These factors remained significant in the multivariate analysis. In the univariate analysis of BOS change, IOP decrease and OPP increase were significantly associated with BOS increase in the temporal ONH, βPPA, and γPPA regions (Tables 3, 4, 5). In multivariate analysis, IOP changes remained significant in all regions. As with other significant variables in the multivariate analysis, an increase in the pulse rate was significantly correlated with BOS increase in the temporal ONH and βPPA regions. The difference in the LSFG measurement time was a significant determinant of BOS changes in the βPPA region.

**Table 2:** Univariate and multivariate analysis of factors associated with MT change of temporal βPPA.

Independent variables	Univariate	Multivariate (IOP, MAP)	Multivariate (OPP)
Time, month	0.048 (0.10), 0.64		
Sex, male versus female	0.28 (0.24), 0.25		
Hypertension	− 0.29 (0.24), 0.23		
No. of baseline antiglaucoma eye drops	0.00073 (0.18), 0.99		
Age, year	− 0.012 (0.014), 0.39		
Baseline axial length, mm	0.045 (0.11), 0.69		
Baseline mean deviation, dB	− 0.055 (0.019), 0.004*	− 0.077 (0.021), 0.001*	− 0.074 (0.019), < 0.001*
Baseline MT, AU	0.033 (0.093), 0.73	− 0.19 (0.10), 0.065	− 0.19 (0.098), 0.047*
IOP change, mmHg	− 0.016 (0.019), 0.41		
MAP change, mmHg	0.025 (0.010), 0.017*	0.026 (0.0096), 0.007*	
OPP change, mmHg	0.033 (0.012), 0.005*		0.038 (0.011), 0.001*
Pulse rate change, beats/min	0.0071 (0.010), 0.48		
Retinal thickness change, μm	0.014 (0.0094), 0.13		
Choroidal thickness change, μm	0.0028 (0.0065), 0.67		
PPA area, mm^2^	− 0.081 (0.68), 0.91		
Difference in LSFG measurement time	− 0.18 (1.02), 0.85	− 0.39 (1.1), 0.71	− 0.77 (1.1), 0.46

Multivariate analysis was performed in two models, one containing IOP and MAP and one containing OPP. The results are listed in the order of coefficient (standard error) and P-value.

*PPA* peripapillary atrophy, *MT* average mean blur rate in tissue area, *IOP* intraocular pressure, *MAP* mean arterial pressure, *OPP* Ocular Perfusion Pressure, *LSFG* Laser speckle flowgraphy.

*P < 0.05.

**Table 3:** Univariate and multivariate analysis of factors associated with BOS change of temporal ONH.

Independent variables	Univariate	Multivariate (IOP, MAP)	Multivariate (OPP)
Time, month	0.48 (0.45), 0.28		
Sex, male versus female	4.9 (2.3), 0.033*		
Hypertension	0.068 (2.4), 0.98		
No. of baseline antiglaucoma eye drops	− 3.5 (1.7), 0.040*		
Age, year	− 0.12 (0.14), 0.39		
Baseline axial length, mm	− 0.31 (1.1), 0.78		
Baseline mean deviation, dB	0.12 (0.21), 0.58		
Baseline BOS, AU	− 0.59 (0.063), < 0.001*	− 0.55 (0.055), < 0.001*	− 0.61 (0.057), < 0.001*
Baseline MT, AU	− 0.18 (0.53), 0.74		
MT change, AU	0.22 (0.42), 0.61	0.33 (0.31), 0.29	0.26 (0.35), 0.45
IOP change, mmHg	− 0.48 (0.12), < 0.001*	− 0.35 (0.089), < 0.001*	
MAP change, mmHg	0.0046 (0.061), 0.94		
OPP change, mmHg	0.18 (0.082), 0.025*		0.21 (0.065), 0.001*
Pulse rate change, beats/min	0.092 (0.052), 0.076	0.096 (0.044), 0.030*	0.11 (0.051), 0.038*
Disc area, mm^2^	− 3.0 (5.7), 0.60		
Difference in LSFG measurement time	0.59 (5.7), 0.92	2.7 (4.5), 0.54	− 2.7 (5.3), 0.62

Multivariate analysis was performed in two groups, one containing IOP and MAP and one containing OPP. The results are listed in the order of coefficient (standard error) and P-value.

*BOS* blow out score, *ONH* optic nerve head, *MT* average mean blur rate in tissue area, *IOP* intraocular pressure, *MAP* mean arterial pressure, *OPP* Ocular Perfusion Pressure, *LSFG* Laser speckle flowgraphy.

*P < 0.05.

**Table 4:** Univariate and multivariate analysis of factors associated with BOS change of temporal βPPA.

Independent variables	Univariate	Multivariate (IOP, MAP)	Multivariate (OPP)
Time, month	1.0 (0.65), 0.11		
Sex, male versus female	7.4 (3.5), 0.038*		
Hypertension	0.053 (3.7), 0.99		
No. of baseline antiglaucoma eye drops	− 4.6 (2.6), 0.078		
Age, year	− 0.28 (0.21), 0.18		
Baseline axial length, mm	− 0.86 (1.7), 0.61		
Baseline mean deviation, dB	0.081 (0.32), 0.80		
Baseline BOS, AU	− 0.67 (0.061), < 0.001*	− 0.64 (0.045), < 0.001*	− 0.68 (0.057), < 0.001*
Baseline MT, AU	− 0.43 (1.4), 0.76		
MT change, AU	2.5 (0.76), 0.001*	1.6 (0.53), 0.003*	1.5 (0.62), 0.014*
IOP change, mmHg	− 0.57 (0.20), 0.004*	− 0.45 (0.11), < 0.001*	
MAP change, mmHg	− 0.0076 (0.064), 0.91		
OPP change, mmHg	0.32 (0.14), 0.023*		
Pulse rate change, beats/min	0.19 (0.079), 0.017*	0.20 (0.059), 0.001*	
Retinal thickness change, μm	0.0063 (0.080), 0.94		
Choroidal thickness change, μm	0.11 (0.047), 0.024*		
βPPA area, mm^2^	14.7 (9.9), 0.14		
Difference in LSFG measurement time	20.6 (8.5), 0.016*	18.2 (5.6), 0.001*	25.1 (6.4), < 0.001*

Multivariate analysis was performed in two groups, one containing IOP and MAP and one containing OPP. The results are listed in the order of coefficient (standard error) and P-value.

*BOS* blow out score, *PPA* peripapillary atrophy, *MT* average mean blur rate in tissue area, *IOP* intraocular pressure, *MAP* mean arterial pressure, *OPP* Ocular Perfusion Pressure, *LSFG* Laser speckle flowgraphy.

*P < 0.05.

**Table 5:** Univariate and multivariate analysis of factors associated with BOS change of temporal γPPA.

Independent variables	Univariate	Multivariate (IOP, MAP)	Multivariate (OPP)
Time, month	0.51 (1.1), 0.66		
Sex, male versus female	7.0 (2.4), 0.003*		
Hypertension	− 0.15 (3.6), 0.97		
No. of baseline antiglaucoma eye drops	− 5.2 (2.5), 0.038*		
Age, year	− 0.24 (0.20), 0.24		
Baseline axial length, mm	− 0.54 (1.6), 0.74		
Baseline mean deviation, dB	0.20 (0.31), 0.52		
Baseline BOS, AU	− 0.67 (0.065), < 0.001*	− 0.59 (0.062), < 0.001*	− 0.61 (0.069), < 0.001*
Baseline MT, AU	− 0.75 (0.54), 0.17		
MT change, AU	2.1 (0.64), 0.001*	1.5 (0.56), 0.009*	1.9 (0.73), 0.010*
IOP change, mmHg	− 0.81 (0.18), < 0.001*	− 0.42 (0.14), 0.002*	
MAP change, mmHg	0.031 (0.11), 0.77		
OPP change, mmHg	0.28 (0.13), 0.035*		0.26 (0.10), 0.011*
Pulse rate change, beats/min	− 0.064 (0.096), 0.50		
Retinal thickness change, μm	0.12 (0.098), 0.21		
γPPA area, mm^2^	− 3.8 (7.9), 0.63		
Difference in LSFG measurement time	− 9.6 (9.4), 0.31	− 3.0 (7.0), 0.67	− 12.2 (8.9), 0.17

Multivariate analysis was performed in two groups, one containing IOP and MAP and one containing OPP. The results are listed in the order of coefficient (standard error) and P-value.

*BOS* blow out score, *PPA* peripapillary atrophy, *MT* average mean blur rate in tissue area, *IOP* intraocular pressure, *MAP* mean arterial pressure, *OPP* Ocular Perfusion Pressure, *LSFG* Laser speckle flowgraphy.

*P < 0.05.

## Discussion

In this prospective study, the blood flow in the temporal ONH and PPA regions was measured using LSFG before and after trabeculectomy. Many previous studies have reported postoperative changes in blood flow in the ONH^11–22^, but no study has investigated such changes by focusing on the PPA regions. Witkowska et al. reported exercise-induced changes in peripapillary blood flow using LSFG. However, the width of the annular peripapillary analytical area was fixed at 50% of the optic disc diameter regardless of the presence of PPA^23^. Kiyota et al. reported a relationship between MT in PPA and central visual field progression^24^. In that study, although LSFG was measured in the PPA region, it was not divided into βPPA and γPPA. Moreover, the measurement area was demarcated by an arbitrary ellipse in the LSFG images that did not exactly fit the shape of the PPA. In the present study, we measured LSFG in βPPA and γPPA separately using OCT and SLO images. Furthermore, we developed a new software that allowed us to precisely set the analytical area for PPA using spline fitting.

In the present study, MT increased in the temporal βPPA region but not in the temporal ONH and γPPA regions after trabeculectomy. Tamaki et al. and Takeshima et al. reported that there was no change in MT in ONH after trabeculectomy, which is in agreement with the results of this study^15,16^. Notably, Tamaki et al. set the measurement area on the temporal side of ONH, which was the same location as this study^15^, while Takeshima et al. measured the MT in the entire ONH area^16^. Thus, MT in ONH may remain constant postoperatively regardless of the measurement location. The peripapillary vessel density changes after trabeculectomy have been measured using OCT angiography in several studies. Kim et al. and Shin et al. reported no postoperative changes in vessel density^18,21^, whereas In et al. reported a postoperative increase in vessel density^20^. Although the βPPA and γPPA regions were not identified in those studies, the differences in the proportion of these PPA regions to the peripapillary area per eye may explain the controversial results.

The differences in postoperative MT changes between the temporal ONH, βPPA, and γPPA may be due to the differences in the blood supply or structural characteristics. The anterior optic nerve can be divided into four regions: the nerve fiber layer, prelaminar, lamina cribrosa and retrolaminar regions^25,26^. A human casting study revealed that the central retinal artery contributes to the blood supply to the superficial region, the nerve fiber layer, while the short posterior ciliary arteries are the main blood supply to the other deeper regions^26^. Notably, Wang et al.^27^ showed that MT in ONH of nonhuman primates is highly correlated with blood flow in the retrolaminar region measured using the microsphere method (R^2^ = 0.88; P < 0.001), where the short posterior ciliary arteries are the main blood supply^26,28,29^.

In the PPA regions, the retinal tissues in βPPA and γPPA receive blood supply from the central retinal artery, and choroidal blood flow in βPPA is derived from the short posterior ciliary arteries^30,31^. The anterior optic disc and choroid receive blood supply from the short posterior ciliary arteries; however, the optic nerve vasculature and choroidal vasculature are largely separate^26,31^. Thus, the ONH, βPPA, and γPPA regions have different blood supplies from each other, which cannot explain the current study results: postoperative MT increases only in the βPPA and BOS increases in all regions. The unique structural characteristics of βPPA from ONH and γPPA are the location outside BMO and the presence of the choroid. Since the macular choroidal blood flow increased after trabeculectomy^11,22^, MT increase in βPPA may reflect blood flow changes in the peripapillary choroid. Moreover, changes in the circulatory parameters (i.e., MAP or OPP), but not IOP changes, were significant factors associated with MT changes in βPPA. Takamatsu et al. reported that MBR changes in the macula after trabeculectomy correlated significantly with changes in MAP or OPP, but not IOP changes^22^. The common feature between βPPA and the macula (i.e., MBR changes associated with changes in circulatory parameters) also supports the significance of choroidal blood flow in βPPA.

In this study, IOP decrease-associated BOS increase was consistently found in the ONH, βPPA, and γPPA regions after trabeculectomy despite considerable differences in the structure between these regions. The retinal thickness in the βPPA and γPPA regions and choroidal thickness in βPPA increased postoperatively. Reversal of optic disc cupping after IOP reduction in adult glaucoma has been reported using stereo photography^32^. More recently, enhanced-depth imaging OCT revealed a significant reduction in posterior displacement and an increase in the thickness of the lamina cribrosa and prelaminar tissue after trabeculectomy^33^. These structural changes due to IOP reduction may relieve vascular transmural pressure and reduce resistance to blood flow, as represented by an increase in BOS. As with other factors associated with postoperative BOS changes, the difference in the LSFG measurement time was significant only in βPPA. On average, the postoperative measurement time was 4 to 5 h earlier than that at the baseline measurement. Earlier measurement timing (i.e., in the morning) compared with the baseline (i.e., in the afternoon) was associated with a smaller BOS increase. Since the choroid has rich autonomic innervation^30^, hemodynamics in the peripapillary choroid may show diurnal fluctuations. Usui et al. reported that the subfoveal choroid in healthy subjects was thicker at night and thinner during the daytime, which correlated negatively with systolic blood pressure^34^. Iwase et al. demonstrated that MBR in the macular choroid of healthy eyes showed significant diurnal variations, with a trough at 15:00 and a peak at 18:00, which was consistent with the fluctuation pattern of the blood pressure parameters^35^. The different patterns of diurnal variation between the choroidal thickness and choroidal blood flow may be related to the different BOS changes in βPPA, depending on the difference in measurement time. Further studies are needed to examine diurnal variations in the structure and hemodynamics of the peripapillary choroid.

Our study has several limitations, including the small sample size and patient characteristics of Japanese patients with open-angle glaucoma. To compare MT changes in ONH, βPPA, and γPPA of the same eye, only the eyes with substantial amounts of βPPA and γPPA were studied. Therefore, the results may not be applicable to eyes without βPPA or γPPA. The retinal or choroidal thickness in PPA was measured in a single B-scan image, which may not represent the entire PPA area.

In conclusion, we investigated the changes in blood flow after trabeculectomy in the ONH, βPPA, and γPPA using LSFG. MT, which reflects blood flow in the tissue area, increased postoperatively only in the βPPA region where the choroid is present. The change in MT in βPPA was associated with changes in the circulatory parameters, OPP, and MAP, which further indicates the significant contribution of choroidal blood flow to MT in βPPA. In contrast, IOP decrease-associated BOS increase occurred postoperatively in all regions, which indicates that IOP reduction may decrease vascular transmural pressure and contribute to stable blood flow uniformly, despite structural differences between the regions. Future studies are warranted to clarify the clinical significance of blood flow changes after trabeculectomy in the temporal ONH region, including βPPA and γPPA, the most critical locations for central visual field damage in GON.

## Methods

### Subjects and study protocol

This prospective study included patients with open-angle glaucoma who underwent trabeculectomy at the Kanazawa University Hospital. The study protocol complied with the Declaration of Helsinki and was approved by the ethics committee of the Kanazawa University. Written informed consent was obtained from all participants.

The details of the study protocol are described in a previous report^16^. Briefly, patients with primary open-angle glaucoma or exfoliation glaucoma were included in this study. Patients who underwent previous intraocular surgery, except glaucoma and cataract surgery, or had a history of IOP-lowering treatment after trabeculectomy were excluded. Eyes with fundus diseases, a long axial length (> 27.00 mm), or small PPA area (βPPA < 0.2 μm^2^ or γPPA < 0.05 μm^2^) were excluded. A trabeculectomy was performed using a fornix-based conjunctival flap. Mitomycin C (0.04%) was used. EX-Press shunts were implanted at the discretion of the surgeon. The blebs were managed using argon laser suture lysis to enhance filtration.

The patients underwent preoperative ophthalmic examinations, including measurements of best-corrected visual acuity, refraction, axial length, and IOP by Goldmann applanation tonometry. Slit-lamp examination, gonioscopy, fundus examination, and visual field tests (Humphrey Field Analyzer; Carl Zeiss Meditec, Dublin, CA, USA) using the 24-2 Swedish interactive threshold algorithm were also performed. Additionally, the systemic blood pressure was measured using an automated sphygmomanometer, and OPP was calculated using the following formula: OPP = 2/3 MAP − IOP, MAP = diastolic blood pressure + 1/3 (systolic blood pressure–diastolic blood pressure). The IOP and blood pressure measurements were performed at baseline and at 1, 3, and 6 months after trabeculectomy, along with LSFG and OCT imaging.

### Measurement of the PPA area

**Figure 3:**
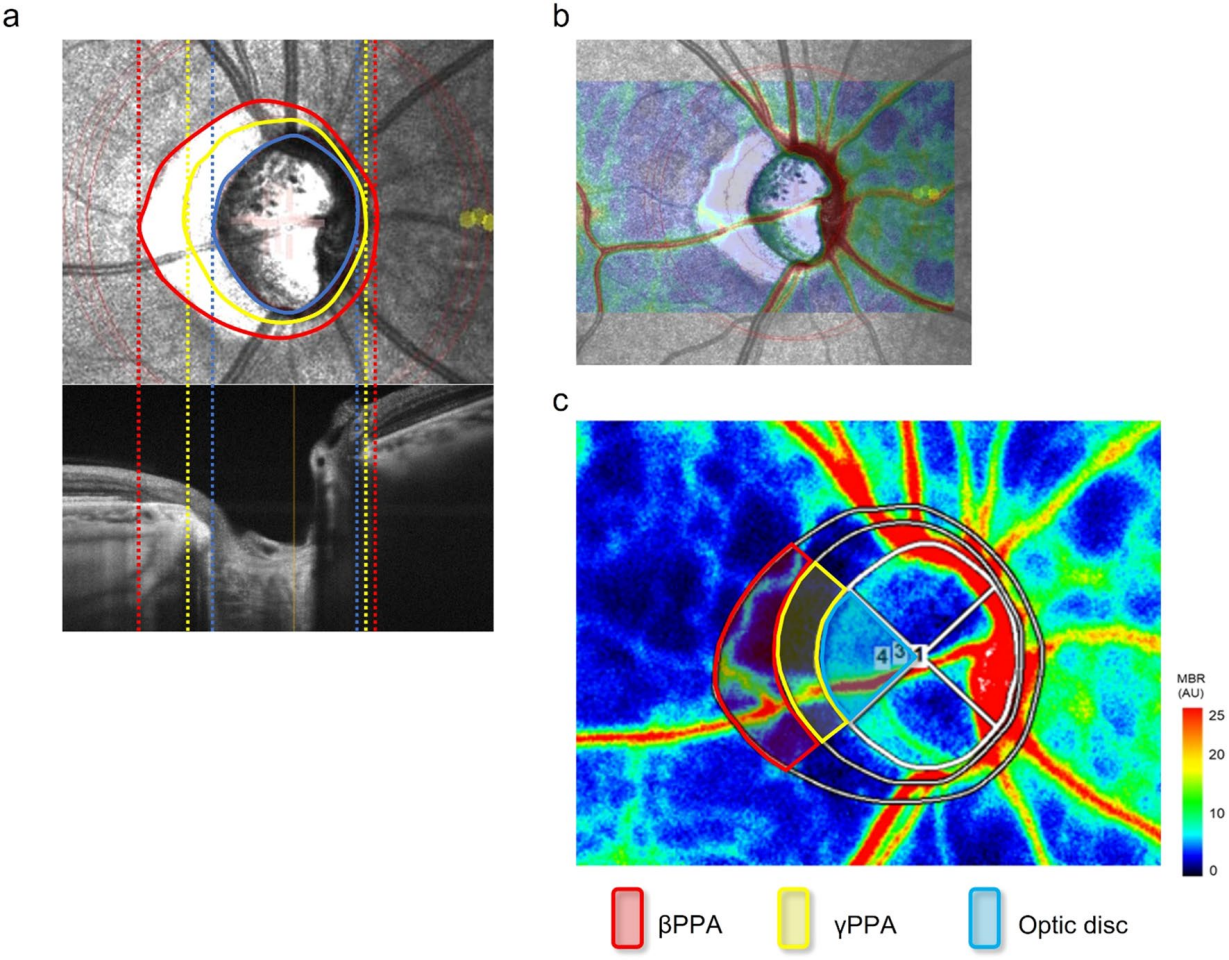
Measurement of area and blood flow of temporal ONH, βPPA and γPPA. (**a**) The built-in OCT software automatically determined the disc margin as the BMO (yellow circle in the SLO image). The disc area was derived by modifying the BMO circle to match the CDM on the SLO image with reference to the fundus photograph presented in another display (blue circle). The outer border of the βPPA was determined by viewing the SLO and B-scan images (red circles). The βPPA zone corresponds to the area between red and yellow circles. The γPPA zone corresponds to the area between the blue and yellow circles. The lower images are the B-scan images along the horizontal white lines in the corresponding SLO images. (**b**) The LSFG and SLO images are overlaid based on the blood vessel positions. (**c**) A rubber band is set by tracing the RPE edge, BMO, and CDM defined in the SLO image. All rubber bands are divided into quadrants of 90° along the superior vs. inferior and nasal vs. temporal axes, centered on the ONH, and waveform parameters in the temporal quadrant of the ONH and PPA regions were analyzed.

The details of the method have been described in our previous study^36^. Briefly, a raster scan of spectral-domain OCT (RS-3000, Nidek Co., Ltd., Gamagori, Japan) was performed over a 6 × 6-mm^2^ area centered on ONH. The built-in OCT software automatically determined BMO as the optic disc margin. We manually corrected the OCT-determined disc area (i.e., the BMO area) as needed by viewing the BMO positions in the B-scan images (Fig. 3a). The γPPA area between the BMO and clinical disc margin (CDM) observed in the fundus photographs, and the βPPA area between BMO and RPE tip were determined by modifying the BMO circle to match the CDM or RPE tip on the SLO image by referring to the fundus photos or B-scan images^9,37^ (Fig. 3a). The βPPA area was derived by subtracting the BMO area from the area inside the outer border of βPPA. The γPPA area was derived by subtracting the clinical disc area from the BMO area. A modified Littmann’s formula (Bennett’s formula) was used to correct for the ocular magnification effect associated with OCT scans in the area measurements^38^. The measurements were made by a well-trained examiner (S. U.) with masking of clinical information. The method had excellent intra- and inter-observer reproducibility, with inter-observer intraclass correlation coefficients of 0.995, 0.961, and 0.949 for the disc, βPPA, and γPPA areas, respectively^36^. The PPA and ONH areas were divided into quadrants centered on ONH, and the values in the temporal quadrant were used for further analysis (Fig. 3c).

### Measurement of retinal thickness and choroidal thickness

Measurement of the retinal thickness and choroidal thickness in the temporal βPPA and γPPA regions was performed with horizontal B-scans using enhanced depth imaging centered on the ONH using ImageJ software (rsb.info.nih.gov/ij). For retinal and choroidal area measurements, the borders of the retina or choroid in βPPA and the border of the retina in the γPPA were manually delineated, and the thickness was derived by dividing the area by the PPA width (Fig. S4). The choroidal thickness and retinal thickness measurements were performed twice by a single rater in a masked fashion, and a third evaluation was performed if the first and second evaluations differed by 100 μm or more and was adopted as the final decision.

### Blood-flow measurement by LSFG

The principles of LSFG have been previously detailed^39,40^. Briefly, the device used was a fundus camera fitted with a standard CCD camera and a diode laser. MBR was determined from the speckle pattern generated by the interference of light reflected from the blood cells in the fundus. It indicates the relative blood flow velocity and is expressed in arbitrary units (AU). The MBR images were obtained over 4 s at a speed of 30 fps. The MBR waveform was delineated by plotting the MBR for each frame using automatic detection at the beginning and end of each heartbeat.

The MBR images centered on the ONH were captured after pupillary dilatation with 0.4% tropicamide. The MBR data were analyzed using an LSFG analyzer (version 3.1.68.2; Softcare Ltd., Fig. 3b,c). The vessel and tissue areas were divided according to an automated definitive threshold. We analyzed the MBR in the tissue area. SLO images indicating the RPE tip, BMO, and CDM were superimposed on the MBR images with aligned blood vessels. Three rubber bands for defining the measurement area were set along the indicated RPE tip, BMO, and CDM, using spline curves. The three rubber bands were further divided into quadrants centered on ONH, and the waveform changes in the temporal quadrants of the ONH and PPA regions were analyzed.

The following MBR waveform parameters were evaluated: the average MBR in the tissue area (MT), the mean MBR of all frames, blowout score (BOS), resistivity index (RI), falling rate (FR), skew, acceleration time index (ATI), and blowout time (BOT) (Supplementary Fig. S5). The average waveforms of MT in the temporal ONH, βPPA, and γPPA were created by averaging MT from all patients after normalizing the heartbeat duration to the range of 0–100% in each patient and were compared among the four measurement time points.

### Statistical analysis

To account for repeated measurements of LSFG, we used a mixed-effects model with eye-specific random effects to evaluate the postoperative changes in IOP, MAP, OPP, choroidal thickness, retinal thickness, and MBR waveform parameters in the temporal ONH, βPPA, and γPPA regions. For MBR waveform parameters that changed significantly after trabeculectomy, the factors associated with postoperative changes were examined by univariate and multivariate analyses, using a mixed-effects model with eye-specific random effects. Since BOS and RI are highly correlated, only the factors associated with BOS changes were analyzed. Multivariate mixed-effects models were created with variables with a P-value of less than 0.2 in the univariate analysis and selected variables. The final model was created by backward elimination using only the variables with P < 0.05. The difference in the LSFG measurement time of the day from baseline and MT change were retained as possible confounders in the models, regardless of the P-values. The OPP calculation formula includes IOP and MAP; thus, mutual interference can occur when performing a multivariate analysis by simultaneously including IOP, MAP, and OPP as independent variables. Therefore, multivariate analysis was performed in two ways: with IOP and MAP or OPP. Statistical analyses were performed using the STATA 15.0 software (StataCorp, College Station, TX, USA). Statistical significance was set at P < 0.05.

## Supplementary Information

Supplementary Information.

## Data Availability

The datasets generated during and/or analyzed during the current study are available from the corresponding author on reasonable request.
