# Supplementary material for: Waveform changes of laser speckle flowgraphy in the temporal optic nerve head and peripapillary atrophy after trabeculectomy in open-angle glaucoma
Source: Sci Rep. 2022 Jun 13;12:9802. doi: 10.1038/s41598-022-13989-2 (PMC9192707; doi:10.1038/s41598-022-13989-2)
Supplement: Supplementary file 1 — Supplementary Information. [file 41598_2022_13989_MOESM1_ESM.docx]

**Supplementary information**

| Temporal ONH |  |
| --- | --- |
| MT (AU) | 6.9±2.3 (2.8 to 13.8) |
| BOS (AU) | 74.1±10.3 (29.8 to 86.2) |
| RI (AU) | 0.38±0.10 (0.22 to 0.75) |
| BOT (AU) | 48.7±4.8 (37.2 to 57.5) |
| ATI (AU) | 34.0±6.6 (25.7 to 63.9) |
| Skew (AU) | 10.7±4.1 (-5.9 to 19.5) |
| FR (AU) | 13.6±1.1 (10.1 to 16.2) |
| Disc area (mm^2^) | 0.52±0.20 (0.14 to 1.2) |
| Temporal βPPA |  |
| MT (AU) | 4.0±1.3 (1.6 to 7.9) |
| BOS (AU) | 68.4±14.4 (3.5 to 83.9) |
| RI (AU) | 0.43±0.13 (0.23 to 0.90) |
| BOT (AU) | 48.8±6.3 (38.1 to 64.1) |
| ATI (AU) | 34.1±6.7 (26.1 to 61.1) |
| Skew (AU) | 10.8±4.5 (-7.7 to 18.5) |
| FR (AU) | 13.9±1.2 (9.8 to 15.7) |
|  |  |
| PPA area (mm^2^) | 0.43±0.20 (0.20 to 0.96) |
| Retinal thickness (μm) | 162.1±32.3 (109.2 to 228.6) |
| Choroidal thickness (μm) | 78.3±36.3 (28.9 to 153.6) |
| Temporal γPPA |  |
| MT (AU) | 5.0±2.3 (1.6 to 10.7) |
| BOS (AU) | 69.7±13.7 (9.5 to 84.7) |
| RI (AU) | 0.42±0.12 (0.25 to 0.87) |
| BOT (AU) | 48.0±7.2 (34.9 to 68.3) |
| ATI (AU) | 32.8±6.1 (23.9 to 60.8) |
| Skew (AU) | 11.4±4.3 (-6.2 to 19.2) |
| FR (AU) | 13.9±1.3 (10.2 to 16.1) |
| PPA area (mm^2^) | 0.31±0.23 (0.054 to 0.79) |
| Retinal thickness (μm) | 136.9±42.0 (67.4 to 234.5) |
| **Table S1. Baseline values of parameters in temporal ONH, βPPA, γPPA.**  PPA peripapillary atrophy, MT: average mean blur rate in tissue area, BOS: blow out score, RI: resistivity index, BOT: blowout time, ATI: acceleration time index, FR: falling rate, | |


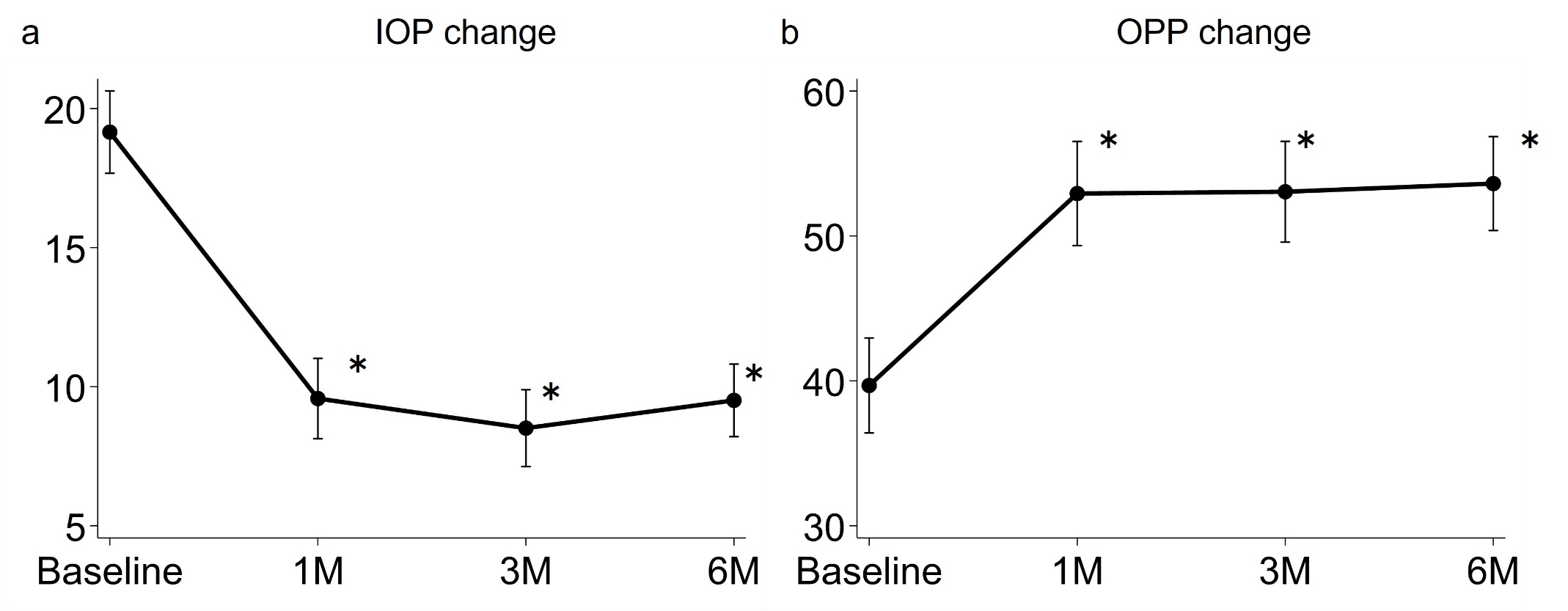


**Figure S1. Changes in IOP and OPP after trabeculectomy.**

a: IOP. b: OPP.*P < 0.05


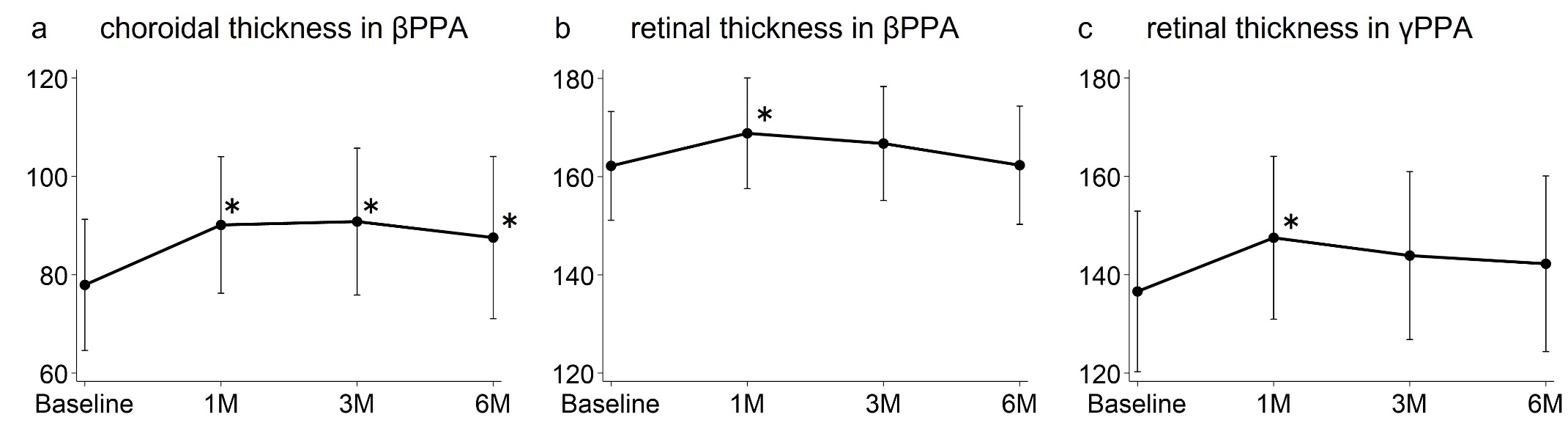


**Figure S2**. **Changes in retinal thickness and choroidal thickness after trabeculectomy.**

a: Changes in the choroidal thickness in the temporal βPPA. b: Changes in the retinal thickness in the temporal βPPA. c: Changes in the retinal thickness in the temporal γPPA. *P < 0.05


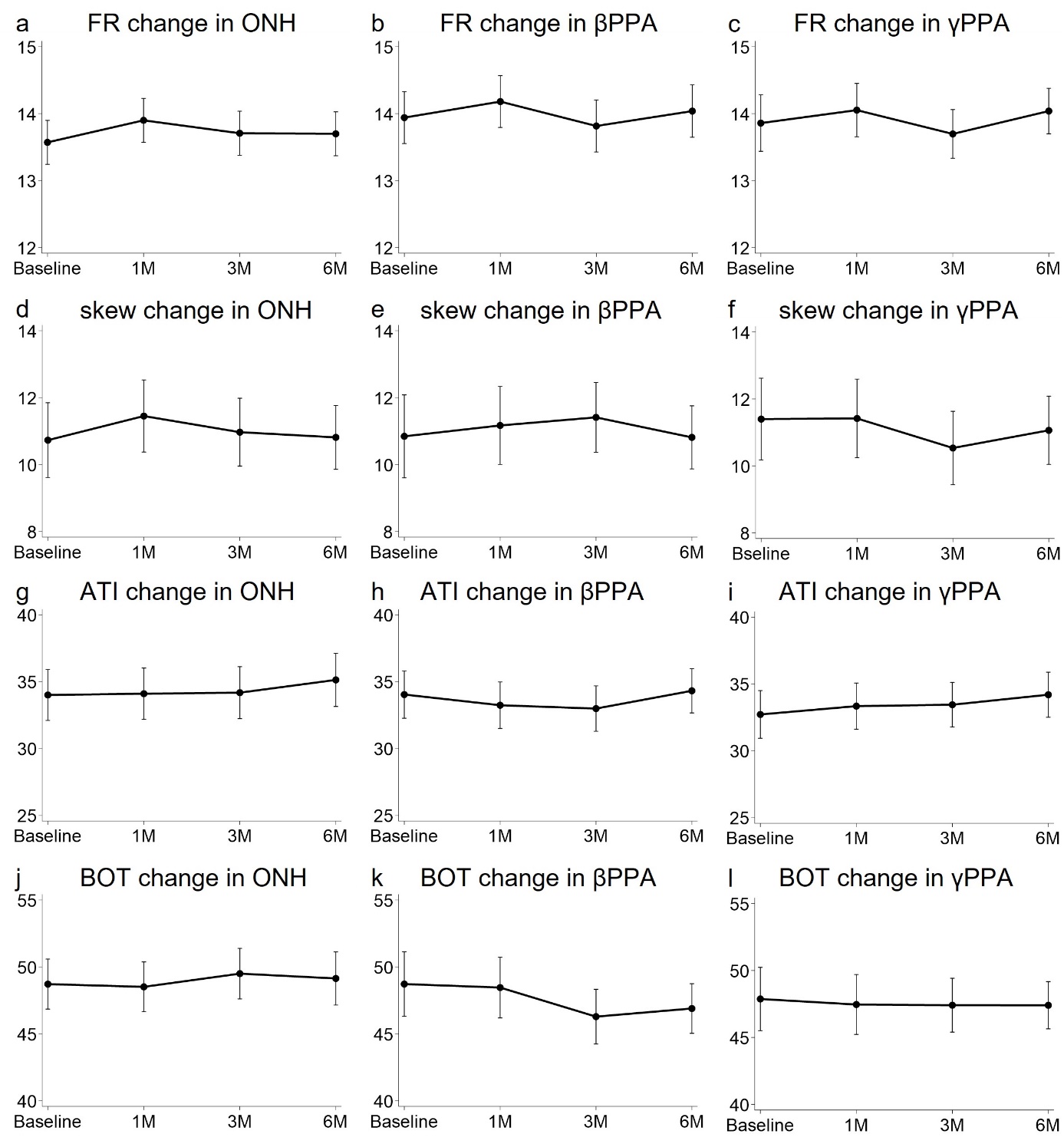


**Figure S3. Change in the MBR waveform parameters in temporal ONH, βPPA, and γPPA after trabeculectomy.**

a, b, c: FR in ONH, βPPA, and γPPA, respectively. d, e, f: skew in ONH, βPPA, and γPPA, respectively. g, h, i: ATI in ONH, βPPA, and γPPA, respectively. j, k, l: BOT in ONH, βPPA, and γPPA, respectively. *P < 0.05

**
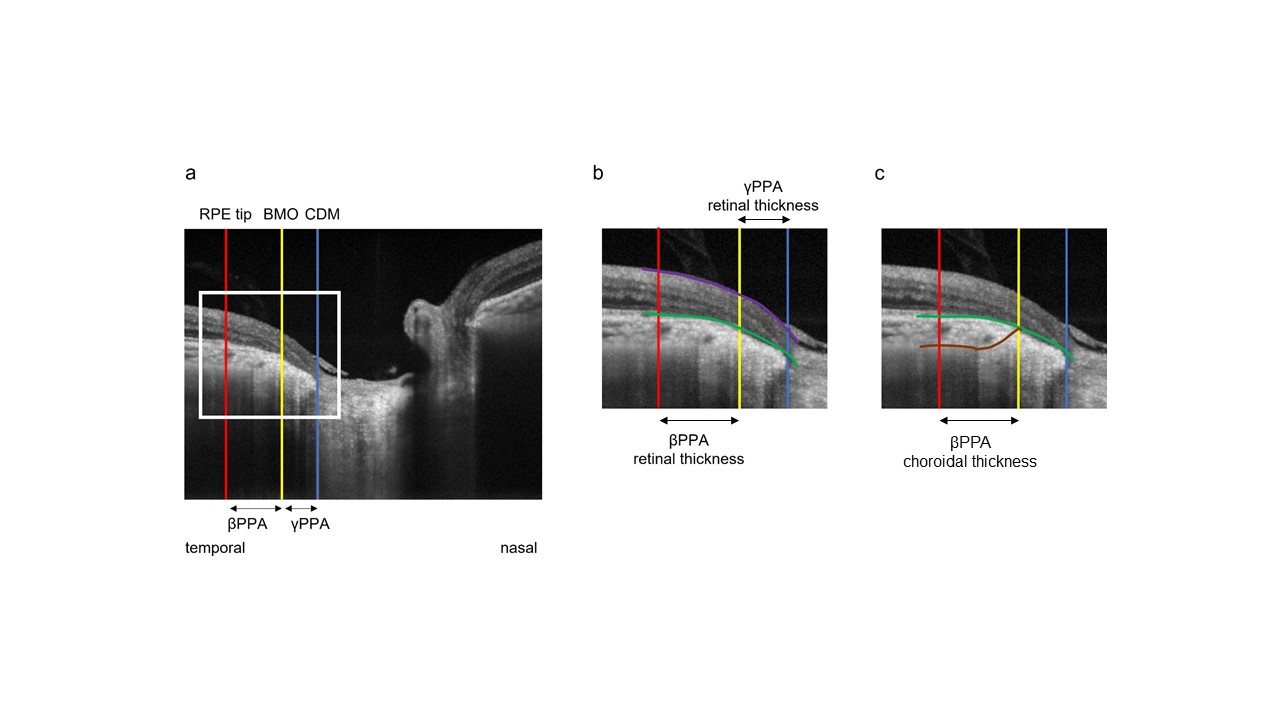
**

**Figure S4. Measurement of the choroidal and retinal thickness.**

a: The area between the RPE end and BMO is defined as βPPA and the area between the BMO and CDM is defined as γPPA. The area within the specified range was measured using ImageJ software. b: The retinal area in the βPPA is surrounded by the internal limiting membrane (purple line), RPE (green line), border of the RPE tip (red line), and border of the BMO (yellow line). The retinal area in the γPPA is surrounded by an internal limiting membrane (purple line), RPE (green line), border of the BMO (yellow line), and border of the CDM (blue line). c: The choroidal area in the βPPA is surrounded by the RPE (green line), posterior boundary of the choroid (brown line), border of the RPE tip (red line), and border of the BMO (yellow line). The retinal and choroid areas were divided by the PPA width (βPPA: between BMO and RPE tip, γPPA: between CDM and BMO), and the mean retinal thickness and choroidal thickness were calculated.
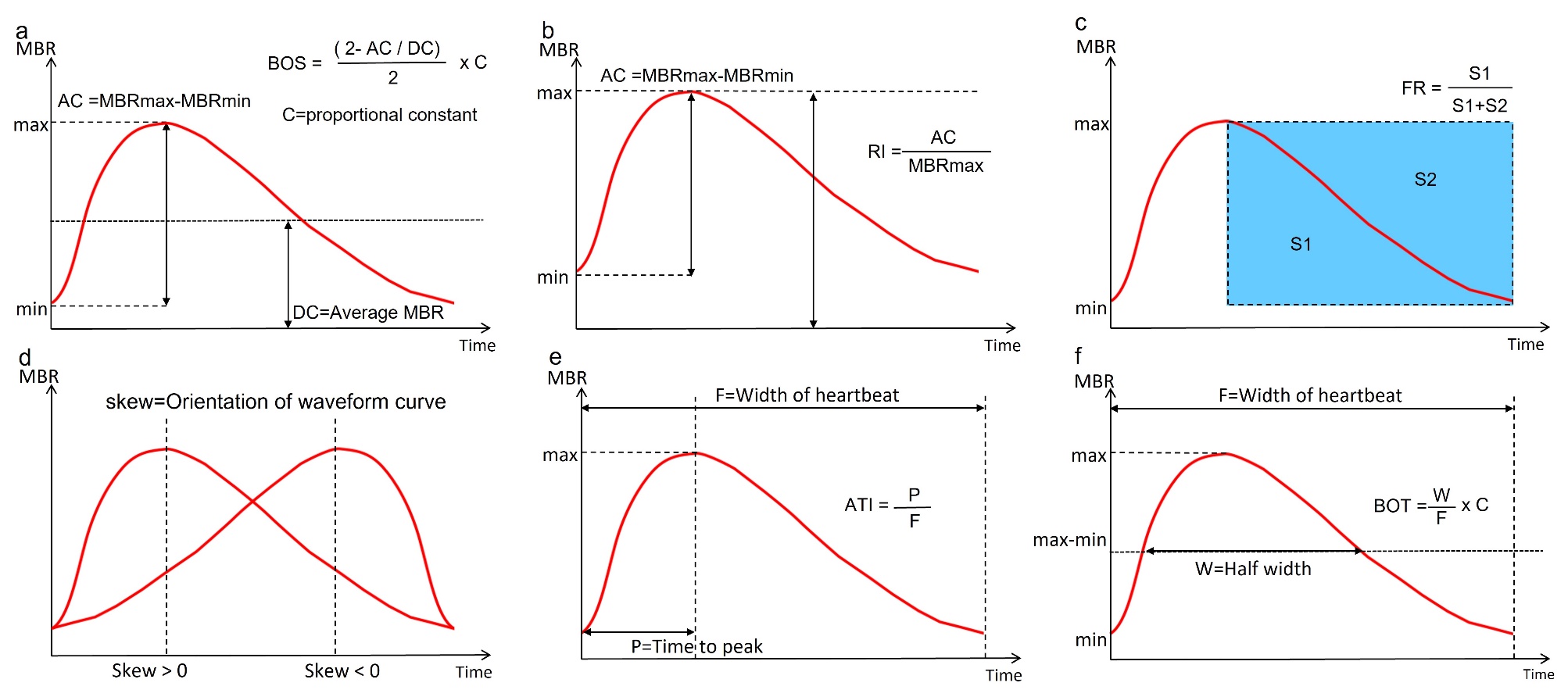


**Figure S5** **Explanation of the MBR waveform parameters.**

a: Blowout score (BOS), indicating blood flow maintained within the blood vessels during each heartbeat. b: resistivity index (RI), obtained by dividing the difference between the maximum and minimum MBR by the maximum MBR. Both BOS and RI are associated with vascular resistance. c: Falling rate (FR) is the proportion of the area of S2 to S1+S2. d: Skew represents waveform asymmetry. A skew value of zero indicates a perfectly symmetrical waveform. If the peak becomes faster and the distribution is leftward, the skew increases; if the peak is slower and the distribution is rightward, the skew decreases. e: Acceleration time index (ATI) is the ratio of the duration of the time to reach a peak (half-width) in one heartbeat (width of a heartbeat). f: The blowout time (BOT) represents the ratio of the half-width of one heartbeat (width of the heartbeat). A high BOT is an indicator of a well-maintained perfusion during the cardiac cycle.
